# How Would Children Register Their Own Births? Insights from a Survey of Students Regarding Birth Registration Knowledge and Policy Suggestions in Kenya

**DOI:** 10.1371/journal.pone.0149925

**Published:** 2016-03-03

**Authors:** Matthew Pelowski, Richard G. Wamai, Joseph Wangombe, Hellen Nyakundi, Geofrey O. Oduwo, Benjamin K. Ngugi, Javier G. Ogembo

**Affiliations:** 1 Faculty of Psychology, Department of Basic Psychological Research and Research Methods, University of Vienna, Vienna, Austria; 2 Department of African American Studies, Northeastern University, Boston, MA, United States of America; 3 School of Public Health, University of Nairobi, Nairobi, Kenya; 4 Department of Statistics and Actuarial Sciences, Kenyatta University, Nairobi, Kenya; 5 Department of Information Systems and Operations Management, Sawyer Business School, Suffolk University, Boston, MA, United States of America; 6 Department of Medicine, University of Massachusetts Medical School, Worcester, MA, United States of America; Centers for Disease Control and Prevention, UNITED STATES

## Abstract

Birth registration and obtaining physical birth certificates impose major challenges in developing countries, with impact on child and community health, education, planning, and all levels of development. However despite initiatives, universal registration is elusive, leading to calls for new approaches to understanding the decisions of parents. In this paper, we report results of a survey of students in grades six to eight (age ~12–16) in an under-registered area of Kenya regarding their own understanding of registration issues and their suggestions for improvement. These students were selected because they themselves were also nearing the age for high school enrollment/entrance examinations, which specifically requires possession of a birth certificate. This assessment was also a companion to our previous representative survey of adults in the same Kenyan region, allowing for parent-child comparison. Results supported previous research, showing that only 43% had birth certificates. At the same time, despite these low totals, students were themselves quite aware of registration factors and purposes. The students also made quite prescient sources for understanding their households’ motivations, with many of their suggestions—for focus on communication of pragmatic benefits, or automatic measures shifting responsibility from parents—mirroring our own previous suggestions, and showing a level of pragmatism not witnessed when surveying their parents. This paper therefore adds evidence to the discussion of registration policy planning. More generally, it also builds on an important trend regarding the treatment of children as stakeholders and important sources of information, and raising an intriguing new avenue for future research.

## Introduction

*“If you want the truth*, *ask a child…”*

—Portion of Danish proverb (Sandheden skal man høre fra børn)

Registration of a child’s birth is one of the more fundamental, far reaching, and troublesome, steps for securing the health and happiness of a society [1]. Registration is noted as a vital, basic human right. It allows children to be counted and acknowledged by a government, and is the first line of protection for shielding children from underage labor, marriage, prostitution, trafficking, and military conscription [2, 3, 4]. Registration, *and increasingly having a physical birth certificate*, is also vital to ensure that children receive access to healthcare or vaccination [5, 6, 7], and is required for many children to enter schooling, travel, gain employment; and therefore is directly tied to their own, and their communities,’ health and future development.

At the same time, despite need to formally recognize and record child births, this is one of the more persistently problematic issues affecting many developing regions [8, 9, 10]. Recent findings by UNICEF [7] estimate that over 30% of the world’s children—56% in sub-Saharan Africa where we conducted the present study—are not registered. Even fewer have birth certificates. Low registration levels have been attributed to a number of reasons, such as lack of infrastructure, transportation, sociocultural factors causing delay or avoidance, or even lack of basic motivation in parents; and has been met with a number of on the ground assessments and implementations [9, 11, 12, 13]. However, despite much effort in study and policy implementations, it is also a well established finding that current approaches have often been disappointingly ineffective at substantially increasing numbers of children registered at birth, or even later in their development [13]. This has led to calls for new answers regarding why children’s births have not been recorded by parents and policy solutions.

Interestingly, one avenue that has not been considered is that of asking children *what they themselves* know about the need, the process and the improvement in certification of their own births. The importance of such study might be framed in several ways. It may be illuminating to discover what children do know about their rights and necessities regarding registration because it does play such an important role regarding their opportunities and health, or if children’s understanding differs materially from that of adults. This fits a growing realization in social and policy research, especially in Western countries, that children have a voice that should be considered [14, 15], as well as findings that children’s own knowledge, or parent-child communication, may play a key role in improving programs related to demographics and health [16]. At the same time, with older children who may soon be specifically impacted by requirements for birth certificates in order to continue with schooling or employment, it may also be enlightening to determine whether they are aware of these impending requirements or of their rights, which also may contribute to policy improvement.

Perhaps most important, children may also be a particular prescient window into the motivations of their parents. In our recent study of parent attitudes in the same region [13], we argued that the current ineffectiveness of registration policy might in fact often be attributed to a disconnect between what parents say in a survey or in studies used for policy planning regarding what impedes them from registering, and their true motivations, which may often be driven more by informed, personal indifference. Parent answers may also be driven by a desire to explain-away previous inaction by producing a list of acceptable factors that absolve the parent. A confirmation bias may also lead parents to believe the difficulties and issues that they list, although these may not bear out objective measurement. This may create a self-fulfilling inability to resolve low registration via policy implementations, which target these same factors. On the other hand, it is a well-regarded truism that children are often surprisingly insightful regarding the motivations and actions of their parents; and often with a candor avoided by adults [17]. To paraphrase a classic truism [18]: Children seldom misquote. More often, they state what parents *should have said* about the issues affecting their households, community, or—potentially—their children’s health.

This is the goal of this paper. We introduce findings from a survey of children’s understanding regarding registration of their births, as well as their ideas regarding policy decisions and motivations of their parents. This was conducted as one aspect of a larger analysis, previously reported in [13], in which we assessed the adult population in an area of rural Kenya, argued to provide a specifically representative example of under-registration of births. We will briefly review the background situation and our previous study findings regarding parent attitudes/answers toward registration of births. We then introduce results from our analysis of these parents’ children, targeting late elementary/junior high age students. We conclude by connecting the findings to the wider adult population and by considering policy suggestions derived from the ideas and awareness of these students. While—it is important to note—this paper does not seek to offer the systematic representative sampling of registration totals, as put forth in our previous work, we hope that the present manuscript can serve as a valuable extension and alternative viewpoint for this topic.

## Review: Kenya and Registration/Certification of Births

Kenya marks an intriguing case for discussion of child registration and birth certificates [13 for review]. On one hand, it possesses many advantages placing it towards the head of the curve for developing nations. Since the founding of the modern state in the beginning of the past century it has enjoyed stable growth [19] and now has a relatively stable democracy and emerging economy as well as infrastructure [20]. These improvements are also supported by a population largely motivated to achieve socioeconomic progress and by a stabilizing government which has enacted multiple reforms in public and private sectors, with the country largely expected to attain middle-income status by 2030 [21]. At the same time, when it does come to registering children, historical rates have been quite low, with issues similar to many other developing areas.

From the founding of the modern country in 1904, to the mid 1980s, registration in Kenya hovered between 30–49% of the population [22]. As the country has moved toward industrialization, there have been attempts to increase registration and issuing of birth certificates. This has included a mix of infrastructural implementations, including educational programs targeting parent awareness, as well as establishment of devoted registration services, and decentralization to the community level [13, 23, 24]. Most notably for the present study, in 2010 the Ministry of Education introduced a requirement that all primary school children must have a birth certificate upon seeking admission to public and private schools, or, especially for those students already enrolled in classes, before registering for national high school entrance examination [25].

Recent totals have shown positive improvement, with roughly 60 percent of children under five now having registered births (2008/09 Kenya Demographic and Health Survey [26]). Especially the tying of registration to entry into formalized schooling has shown recent anecdotal impact [13]. However, totals are still far below the “universal” target set by the government. Even more, only 24 percent of children had birth certificates [26]. This is often attributed to the continuing existence of a large percentage of the population outside of major urban centers [27], contributing to a widening gap, ~10–20 percentage points lower than more urban regions [26, 28]. The pairing of low certification with demands by government for school documentation has also had a secondary impact on children in these areas, delaying or precluding some from continuing studies or from securing employment [13].

In 2010, the government therefore created a new program for Universal Birth Registration (UBR), targeted to the Millennium 2030 program. In this project, the Civil Registration Department partnered with Plan (http://plan-international.org/), one of the world’s largest child centered community development organizations, to undergo an initiative aimed at increasing registration through enhancing accessibility, efficiency and community awareness. This also had the goal of targeting previously under-registered areas by surveying stakeholders with the goal of identifying what factors had impeded registration or certification of births.

### Kwale, Kenya: findings from a representative study of parents

**Fig 1:**
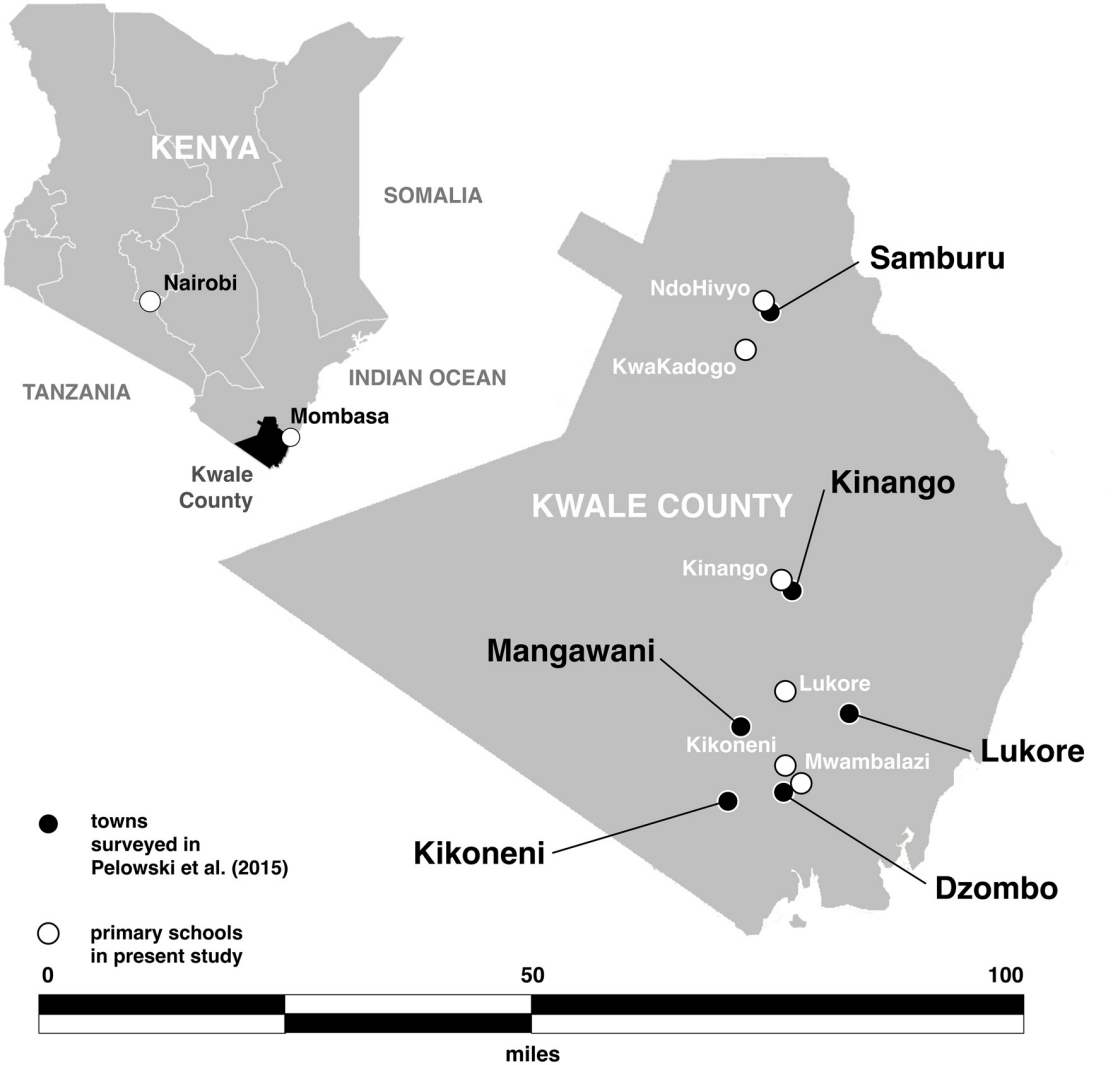
Kwale county Kenya. School testing sites for present study denoted by white circles; towns used in previous testing of adult population [13] denoted by black circles.

The above program served as the basis for our previous study [13], and will also set the context for the present assessment. In collaboration with the Kenyan government and Plan Kenya we conducted a representative sampling (between November 2011 and March 2012) of the adult population at six test sites spread across a region of Kenya specifically selected by the government and Plan as typifying the previous issues with UBR. The designated region, Kwale County, lies in the Southeastern coastal portion of Kenya on the Indian Ocean, bordering Tanzania to the South, and near to the coastal city of Mombasa to the North. The area is a center for small scale farming with a population of roughly 650,000 people (KNBS, 2010), divided into six wards (Fig 1) with a mix of tribal and ethnic groups typical for many regions in Kenya [29]. The area was selected by the government and Plan Kenya because it also has one of the lowest registration rates in the country (37.3% in 2012) [26], and was thus argued to provide an important basis for planning future policy.

This survey revealed birth registration levels even lower than government estimates for the same Coast Province region [26], with 46.5% of parents claiming to have registered all of their children and 7.9% reporting that all children had birth certificates. In addition, we found a number of infrastructural or socioeconomic factors which had significant impact on registration. These included parent education, occupation, rural vs. urban environment, and understanding of the registration process itself. This was coupled with other factors—cost, travel, lack of awareness—mentioned by many parents as reasons why they had not registered, but which, upon further investigation, did not appear to have a significant impact on actual registration action. In fact, as noted above, a key finding was that parents did appear to have sufficient information, resources, and access to successfully register children. Rather, we argued that the most persuasive impediment appeared to be a conscious weighing by parents of perceived benefits. This was coupled with a decision not to take immediate action with registering young children because they did not perceive reasons sufficient to outweigh the small costs. This outcome was then in turn also tied to school registration, which was noted by many parents to be a main impetus for finally taking action when children were close to entering secondary schooling. However, this led to a gap from infancy to junior high where children were not registered.

We concluded that new approaches were needed to motivate, or to modify, behavior of parents. For example, we suggested tying registration to other programs such as vaccination, which would provide an immediate reward to parents. Even better, we advocated avoiding the need for parent decisions via implementation of hospital birth or other top-down initiatives, whereby representatives (such as hospital staff or government) took responsibility of registration from parents, and with the argument that, by better understanding parent psychology rather than merely focusing on limitations, we may find meaningful improvement.

## Study Design and Methodology

The present study builds from the above findings, focusing on the children of the previously surveyed parents. The study was administered between November 2011 and March 2012 to a convenience sample of all students in three grades (six-eight) in six schools within the same Kwale region (Fig 1). Schools were selected from the target region with the goal of assessing those areas previously surveyed in the adult population, while also achieving geographic distribution across all six wards of Kwale. Grades six to eight (roughly ages 12–16) were selected in order to target students who might be more aware of the activities and issues in their community, as well as those who would be specifically preparing to take the entrance examinations for high school following grade eight. This age (11+) has also been shown to align with individuals who have moved beyond earlier cognitive development stages, which can impact understanding or responding to survey questions [17, 30].

To administer the survey, an introduction letter was sent to the schools through the respective District Education Offices to inform head teachers of our project and to request support and permission. A consent letter was also sent to parents to get their permission to survey their children. Before taking the survey, students were informed regarding the study purpose, and their written consent was obtained (see Ethics for more details). Following consent, surveys were conducted with each student by a trained surveyor. These individuals were selected from among volunteers working within each community and instructed by the study authors in data collection. Surveyors visited each school on an appointed day and met with students, working in small groups, with the surveyor reading the questions and students independently writing answers. Questionnaires were originally written in English, however survey questions were also offered in Kiswahili, and students were allowed to answer in the language they found most comfortable. Students were also checked with each question regarding their understanding, and were given unlimited time to make responses. These procedures, as well as the avoidance of negatively formulated questions or overly large number of possible choices, followed suggested best practice for surveys of children [15, 17, 31]. Surveys took about twenty minutes.

### Survey questions and Goals

The survey consisted of a paper-based list of questions divided into four main sections: (1) first this included a number of questions eliciting basic demographic information, including age of students and grade level. This was followed by (2) a section assessing students’ possession of a birth certificate as well as their awareness of the time and conditions whereby it was received. (Note that we targeted possession of an actual certificate rather than the more general question of birth registration because of its importance for future school enrollment). This also included a question relating to awareness of the possession of certificates among siblings and peers, followed by (3) a section assessing student knowledge of purpose and procedures for birth registration/birth certificates. Based on these questions we were interested in assessing what were the basic rates of registration/certification among students, especially those preparing for the high school entrance examination, and if they differed materially from our adult data. We were also interested in students’ awareness of the purpose and procedures for registration in order to determine whether any notable trends could be discovered among answers and possession of a certificate. Finally, (4) we included two questions eliciting students’ own ideas regarding what government or others could do to increase rates of registration/certification in the students’ region as well as how we might motivate their parents.

### Ethics Statement

All studies were approved by the Northeastern University Institutional Review Board and Plan Kenya Nairobi before being conducted. As noted above, the studies made use of an informed consent procedure for parents, school officials, and the individual students. All parties were made aware of the study purpose, procedures, as well as the intended use of the data. Both students and parents were also informed that the answers would be kept anonymous, and that the study was completely voluntary, could be stopped at any time, and that participation would have no bearing on class grading or other consequences. Written consent was obtained from all parties before beginning.

### Statistical analysis

Statistical analyses were carried out with SPSS (IBM corp., version 22.0). Categorical data were summarized using frequency counts and percentages. Continuous data was assessed using means.

Comparison between survey sites (individual schools) showed no significant differences in demographic or grade level distributions, thus all sites were combined in the following analyses. Because the individual schools can be considered to represent different communities, however, which may have meaningful differences in their responses to birth certification, we have also provided between-school comparisons. These were analyzed via univariate Chi square tests. Results are reported in the accompanying tables. Factors affecting possession of a birth certificate were also first analysed by univariate Chi square tests, followed by a binomial logistic regression model, with possession of a certificate as the dependent variable and all factors that had been significant in the univariate analyses included as predictors. In line with the exploratory nature of this study, significance level was set to 5% (two sided) and we performed neither adjustment for multiple testing nor imputation of missing values. We also stress again that, given the convenience sampling technique used for the study, results should primarily be considered as articulating factors impacting this specific group of children. For broader inference-making regarding specific totals, we direct readers to our previous paper [13].

## Results

All students (and their parents) within the contacted classes agreed to participate, resulting in a final dataset of 503 (239 female; 264 male) out of an estimated 15,780 total households within the region (~3.1%) [13]. Mean age of students was 14.6 years (*SD* = 1.9). Students were also evenly distributed among the three grade levels: grade six (31.6%, *n* = 159), seven (33.8%, *n* = 170), and eight (34.6%, *n* = 174). The majority of participants were Muslim (58.1%), followed by Protestant (31.6%) and Catholic (10.3%).

Rates regarding possession of a birth certificate are shown in Table 1. Overall, 42.9% of students said that they possessed a certificate, while 6% did not know the answer. When students who had positively answered the above question were asked when they had received the certificate, half of the respondents (50.5%) clustered in the most recent three year period of 2012 to 2010—or when children were on average 11.6 to 13.6 years of age—while 15.7% received the certificate before 2009 and the remainder did not know. Of those children with siblings who were less than 18 years old (86.1%, *N* = 433, Mean number of siblings = 3.5, *SD* = 1.9), 33.9% were positive that their siblings all had certificates, while 4.4% were not sure of the answer.

**Table 1:** Possession of birth certificate among students, Kwale Kenya (% answer distribution).

		Individual schools^a^						
	All	Lukore	Kikoneni	Kinango	Mwambalazi	NdoHivyo	KwaKadogo	Site comparison
	(*N* = 503)	-100	-93	-75	-85	-75	-75	*X*^2^ (*df*, *N*) ^b^
**Have birth certificate?**								
Yes	42.90%	49	29	74.7	37.6	50.7	18.7	67.9 (10, *N* = 503)***
No	51.1	41	63.4	24	55.3	45.3	77.3	
Don't know	6	10	7.5	1.3	7.1	4	4	
**When did you get certificate?** ^c^								
2012	19	22.4	11.1	17.9	28.1	7.9	35.7	39.1 (25, *N* = 216)*
2011	22.2	34.7	3.7	21.4	21.9	18.4	28.6	
2010	9.3	10.2	7.4	7.1	3.1	18.4	7.1	
2009	2.3	2	0	5.4	0	2.6	0	
Before 2009	15.7	12.2	29.6	21.4	9.4	10.5	7.1	
Don't know	31.5	18.4	48.1	26.8	37.5	42.1	21.4	
**Siblings have birth certificate?** ^d^								
Yes	33.9	33.7	29.1	66.2	10	49.2	17.5	67.7 (10, *N* = 433)***
No	61.7	60.2	65.1	30.9	85.7	50.8	76.2	
Don't know	4.4	6	5.8	2.9	4.3	0	6.3	

*Notes*: All respondents were elementary school age children in grades six to eight. Percentages account for individuals who did not answer specific questions. Answer rate for all questions was > 97%.

^a^ Comparison between survey sites (individual schools) showed no significant differences in demographic or grade level distributions. Provided for information purposes only.

^b^ * and *** indicate statistical significance at the .05 and .001 levels, respectively. Comparisons assessed via Chi square. First number in parentheses refers to degrees of freedom. Second number indicates total sample size.

^c^ Question addressed to only students who answered ‘yes’ to having a certificate (*N* = 216).

^d^ Question addressed to only students who answered ‘yes’ to having a sibling (*N* = 433). Average number of siblings = 3.5 (*SD* = 1.9).

### Knowledge of certificate importance and procedures

As shown in Table 2, when asked to explain the definition of a birth certificate, the majority (61.5%) reported that a birth certificate is a document showing vital statistics or birth information of a child. This was followed by 17.7% who said that it was a document used for school exam registration, and then by a number of other answers (a document used in the “future,” identification (ID) or citizenship, healthcare) all < 10%. When asked whether having a certificate was necessary, 93.2% said ‘yes’. When asked why one needed a certificate, most (31.4%) said that it was required by law, followed by necessity for being recognized as a citizen, and that it was needed for school and exam registration. In response to how soon a child should get a certificate, answers were spread rather evenly among 0–4 months (the correct answer via current Kenyan regulations), before one year of age, and after one year.

**Table 2:** Understanding of birth certificate purpose and procedures among students. Kwale, Kenya.

		individual schools ^a^						
	All	Lukore	Kikoneni	Kinango	Mwambalazi	NdoHivyo	KwaKadogo	site comparison
	(N = 503)	(100)	(93)	(75)	(85)	(75)	(75)	*X*^2^ (*df*, *N*) ^b^
**What is a birth certificate?** ^c^								
Doc. with vital/birth statistics	61.50%	51.0	67.7	68.0	42.4	77.3	64.0	126.0 (30, *N* = 503)***
Doc. for school registration	17.7	17.0	16.0	17.3	23.5	13.3	16.0	
Doc. used in future	6.3	7.0	1.1	1.3	22.4	4.0	0	
Identification (ID)/citizenship	5.1	14.0	1.1	10.7	3.5	5.3	2.7	
Used for healthcare	1.2	5.0	0	0	1.2	0	0	
Other	3.8	5.0	7.4	2.7	7.1	0	13.3	
Don't know	4.2	1.0	6.4	0	0	0	4.0	
**Why do you need a cert.?**								
Required by law	31.4	23.2	49.5	25.3	49.4	12.0	26.7	116.9 (20, N = 500)***
To be recognized as citizen	27.2	32.3	30.8	32.0	18.8	21.3	28.0	
School examination	21.7	19.2	16.5	38.7	18.8	17.3	22.7	
Identification	17.7	23.2	2.2	1.3	11.8	48.0	22.7	
Don't know	0.8	2.0	1.1	2.7	1.2	1.3	0	
**How soon should you get cert.?**								
0–4 months	31.1	36.0	25.8	18.7	42.4	40.5	21.6	28.9 (15, *N* = 501)*
Before one year old	29.1	25.0	37.6	34.7	29.4	21.6	25.7	
After one year	29.7	29.0	24.7	37.3	22.4	31.1	36.5	
Don't know	10.0	10.0	11.8	9.3	5.9	6.8	16.2	
**Where can you get certificate?**								
Chief/Assistant chief	43.3	51.0	26.9	38.7	40.0	37.3	68.0	107.4 (25, *N* = 503)***
Community health worker	19.5	19.0	21.5	17.3	18.8	25.3	14.7	
Nurses/doctors	14.7	17.0	31.2	4.0	16.5	9.3	5.3	
Birth attendants	11.1	4.0	4.3	26.7	8.2	25.3	2.7	
Volunteer organizations	7.0	5.0	9.7	10.7	10.6	1.3	4.0	
Don't know	3.4	4.0	6.5	2.7	5.9	1.3	5.3	
**Who responsible for getting cert.?**								
Parents/relatives	31.4	23.0	12.9	26.7	12.9	62.7	60.0	156.3 (35, *N* = 503)***
Hospital	19.3	18.0	21.5	18.7	23.5	12.0	21.3	
Church/mosque	16.3	17.0	30.1	5.3	29.4	6.7	4.0	
Government/chief	10.6	15.0	7.5	14.7	8.2	8.0	9.3	
Midwife	10.3	10.0	15.1	12.0	14.1	5.3	4.0	
Community organization	5.0	5.0	8.6	16.0	7.1	0	0	
School	3.4	5.0	4.3	4.0	3.5	1.3	1.3	
Don't know	1.4	7.0	0	2.7	1.2	4.0	0	
**Where did you get information?** ^d^								
Chief	32.0	39.8	10.2	55.4	12.0	33.1	26.6	151.7 (40, *N* = 610)***
School	26.4	17.7	26.6	1.4	33.7	15.3	45.6	
Hospital	23.9	18.6	29.7	29.7	16.3	20.2	10.1	
Family	16.3	12.4	12.5	13.5	16.3	20.2	7.6	
Community	5.2	0.9	9.4	0	10.9	2.4	2.5	
Siblings' birth	3.9	2.7	5.5	0	4.3	4.0	5.1	
Church/Mosque	2.6	4.4	2.3	0	3.3	1.6	0	
Midwife	2.4	1.8	3.1	0	2.2	3.2	1.3	
Don't know	0.9	1.8	0.8	0	1.1	0	1.3	
**Know other children with cert.?**								69.2 (5, *N* = 503)***
Yes	61.6	64.0	47.3	61.3	34.1	85.3	84.0	
No	38.3	36.0	52.7	38.7	65.9	14.7	16.0	

*Notes*: All respondents were elementary school children in grades six to eight. Percentages account for individuals who did not answer specific questions. Answer rate for all questions was > 97%.

^a^ Comparison between survey sites (individual schools) showed no significant differences in demographic or grade level distributions. Provided for information purposes only.

^b^ * and *** indicate statistical significance at the .05 and .001 levels, respectively. Comparisons assessed via Chi square. First number in parentheses refers to degrees of freedom. Second number indicates total sample size.

^c^ Notable answers in ‘Other’ category included: general physical description, general notion of "importance," or use for securing employment (all < 1%).

^d^ Respondent could give more than one answer.

When asked where one could file for or receive a certificate, the most noted response was chiefs/assistant chiefs—the community level representatives of local government. This was followed by community health workers, nurses/doctors, or hospital birth attendants. Regarding the individual who should take responsibility to ensure that a child gets a birth certificate, 31.4% noted parents or relatives, 19.3% said hospitals, followed by churches/mosques, or some portion of local/national government. Regarding where they had received their information, the highest number mentioned chiefs (32%), followed by schools, hospitals, and relatives or parents. Over half (61.6%) knew other children who had certificates.

### Factors significantly associated with possession of birth certificate

We then looked at the question of which of the above factors had significant ties to a student’s possession of their own birth certificate. Results of the Chi square analyses, are shown on the left side of Table 3. Among the population of respondents who had answered either ‘yes’ or ‘no’ to having a certificate (n = 473), differences were found between grade levels, with the highest rates of certification found among students in class eight. Differences were also found between religions, with Muslim students showing lower rates, as well as between those who reported that their siblings did/did not have their own certificates. Notably, we found 70.3% certificate possession for those whose siblings also had certificates versus 33.6% for those whose siblings did not.

**Table 3:** Knowledge and demographic factors significantly tied to possession of birth certificate among students in Kwale, Kenya. Univariate analyses (left) and Binomial Logistic Regression (right).

	% have certificate	Univariate Analyses	Binomial Logistic Regression ^a^		
		*X*^*2*^ *(df*, *N)*	Wald ^b^	Odds ratio ^c^	CI (95%)
**Grade level**		36.2 (2, *N* = 473)***	40.7***		
eight	63.9		----	1	----
seven	32.9		36.9***	0.17	[0.10–0.30]
six	38.6		22.8***	0.24	[0.13–0.43]
**School**		58.9 (5, *N* = 473)***	29.3***		
Kinango	75.7		----	1	----
Lukore	54.4		4.6*	0.41	[0.18–0.93]
NdoHivyo	52.8		5.1*	0.38	[0.16–0.88]
Mwambalazi	40.5		4.3*	0.36	[0.14–0.94]
Kikoneni	31.4		8.3**	0.26	[0.11–0.65]
KwaKadogo	19.4		27.6***	0.08	[0.03–0.21]
**Sibling has certificate?**		56.5 (1, *N* = 473)***	38.0***		
Yes	70.3		----	1	----
No	33.6		----	0.2	[0.12–0.34]
**Religion**		9.8 (2, *N* = 473)**	4.3		
Catholic	54.2				
Protestant	53.9				
Muslim	39.5				
**Know other children with cert.?**		5.0 (1, *N* = 473)*	1.9		
Yes	49.7				
No	39.1				
**Definition of cert.**		16.7 (6, *N* = 473)*	4.4		
Doc. of ID/citizenship	83.3				
Doc. used for healthcare	50.0				
Doc. with vital/birth statistics	45.4				
Doc. used in "future"	46.7				
Other (general card or important)	38.9				
Doc. for school/exam registration	36.9				
Don't know	45.0				
**Source of information**		14.0 (7, *N* = 473)*	5.1		
Relatives	56.0				
Church	55.6				
Chief	51.7				
Hospital	48.5				
Midwife/ sibling birth	40.0				
School	37.0				
Community	34.1				
Don't know	23.5				

*Notes*: All respondents were elementary school students in grades six to eight.

^a^ Regression model (right side) performed on all factors shown in left column which had shown significance in individual univariate analyses. All factors treated as categorical. Model significant at *p* < .001. χ2 (24, *N* = 473) = 165.97, Nagelkerke *R*2 = .40, correctly predicted cases = 74.2%.

^b^ *** significant at p < .001, ** p < .01, * p < .05.

^c^ Odds ratios and CIs are shown for significant factors only, and show comparison to topmost category in group.

Odds ratios should not be interpreted as approximated relative risk. Estimated odds as shown in the Table will be closer to 1 than the ratio change of all odds (which cannot reliably be estimated via this approach)

Among knowledge and awareness questions, significant difference was also found regarding definition of a birth certificate. Notably, students who answered that certificates were primarily a “document used for ID (government identification) or citizenship” possessed certificates at the highest rate (83.3%), whereas 36.9% (lowest) of those who answered that certificates were primarily “documents used for exam or school registration” themselves had certificates. We also found significant difference regarding primary source of information. Here, the prominent difference appeared to be use of schools or community, with students using these avenues reporting low rates of certificate possession, compared to higher rates certificate possession among students mentioning hospitals, chiefs, churches and relatives/parents. Those who did not know any source in turn showed the lowest rates of certificate possession (23.5%). Follow-up comparison of source of information and ability of students to give correct information about certificate definition/procedures, did not reach significance. Significant difference was also found for those who knew other children with certificates versus those who did not.

We also found significant difference between schools. However again, univariate Chi square comparisons showed no demographic differences that would explain these patterns. Comparison for knowledge and understanding questions between schools did show significant differences. These are reported in the right sides of Tables 1 and 2. Here as well, patterns were not overly instructive. For example, regarding purpose of a certificate, students from Kinango—with the highest percentage of students possessing certificates—answered that certificates are used to register for school at a relatively higher rate than many other schools. Whereas students in schools with lower percentages of students possessing certificates (e.g., Kwakadogo and Mwambalazi) were more likely to say that a certificate is used for ID. These patterns did not hold for other schools, while essentially the same patterns were found for all schools for other answers, regardless of percentage of students possessing certificates. Significant difference between schools was also found for knowing others who had a certificate. Here again however, Kwakadogo, for example, with the lowest percentage of students with certificates, showed an answer of ‘yes’ to knowing others at the second highest rate (84%), while Kinango (highest percentage of students with certificates) showed relatively low rates of knowing others with certificates.

The one factor that did appear to show both a significant and a *meaningful* pattern of differences was again source of information. Students from schools with the lowest percentages of individuals possessing certificates mentioned schools as their primary information source—Kwakadogo (45.6% of time), Mwambalazi (33.7%). This compared to lower mention of schools as an information source among students at schools with higher percentage of students possessing certificates (e.g., Kinango, 1.4%; Ndohivyo, 15.3%). On the other hand, mention of chiefs was lower among schools whose students showed lowest rates of possessing certificates—Kikoneni, Mwambalazi—compared to schools with higher percentages of students in possession of certificates (see Table 2).

Finally, to better understand the combined contribution of the above factors, we conducted a binary logistic regression. This again used all of the factors (listed on the left side of Table 3) which had shown significance in regards to possession of birth certificates as predictor variables (treated as categorical data). The model results are reported on the right side of Table 3, with Odds Ratios and 95% Confidence Intervals also shown for factors that were found to be significant in the model (indicated by asterisks accompanying the Wald statistics). The decision was made to use a logistic regression and Odds Ratios (ORs), rather than prevalence rates or relative risk (RR), following suggestion [32, 33] that in non-controlled sampling cases where there is high outcome prevalence (above 10%), ORs are a more appropriate summary measure with a more tenable assumption of homogeneity across all individuals in a sample population. Note therefore that the estimates should not be interpreted as approximated risk.

The model (*p* < .001, *χ*^*2*^ [24, *N* = 473] = 165.97, Nagelkerke *R*^2^ = 40%, correctly predicted cases = 74.2%) indicated that only three factors—grade level, school, and whether or not one’s sibling had a certificate—remained significant predictors. Odds ratios indicated that students in grade eight had significantly higher odds of possessing a birth certificate when compared to lower grade levels, while having a sibling with a certificate was associated with higher likelihood of the student him/herself possessing a certificate.

### Students’ suggestions for improving certification of their own births

Students’ own ideas for improving certification rates are reported in Table 4. Regarding how the government could improve registration, student answers—which were given in a free style with no prearranged answer choices—showed a rather even distribution between the themes of (1) direct action, (2) infrastructure and (3) education. The most often mentioned suggestion (16.7% of students) was that the government should in some way take up the full responsibility from parents and therefore complete the process automatically while minimizing parent involvement. This typically involved the suggestion that the government dispatch some official representative (chief or other National level official) to visit children or parents in order to complete the registration process. Following this, 15.2% said that the government should provide more education or information for parents, and 12.7% said that the government should do more to enforce the existing laws or make official checks at schools or villages to ensure that all children were registered; 11.6% also noted that the government should give *non financial* assistance through helping with transportation or paperwork.

**Table 4:** Students’ suggestions for how to improve birth registration and tie to possession of own certificate, Kwale, Kenya.

	% total respondents ^a^	% who possess certificate	*X*^*2*^ Answer x cert. possession
			(*df*, *N*) ^b^
**What should government do to raise birth registration?**			
Register automatically instead of parents.	16.7%	36.7	21.2 (8, *N* = 473)**
Improve education about registration	15.2	50.0	
Enforce the law.	12.7	62.7	
Assist parents (non financial).	11.6	38.2	
Increase hospital births.	8.7	65.9	
Improve access or ease of registration.	7.8	43.2	
Reduce cost.	5.3	32.0	
Don't know.	7.4	34.3	
**How would you encourage your parents to register your birth?**			
Explain importance for school/exams.	32.1	40.1	18.1 (8, *N* = 473)*
Explain importance (general).	20.3	58.3	
Ask third party to talk to them.	9.7	56.5	
Explain importance for ID/citizenship.	8.7	29.3	
Take action: do the registration for them.	7.8	51.4	
Explain importance for getting job.	4.9	30.4	
Explain importance for following law.	1.7	37.5	
Don't know.	9.5	42.2	

*Notes*: All respondents were elementary school students in grades six to eight.

^a^ Percentages account for individuals who did not answer specific questions. Answer rate for all questions was > 97%.

^b^ * and ** indicate statistical significance at the .05 and .01 levels, respectively.

Comparisons assessed via Chi square. First number in parentheses refers to degrees of freedom. Second number indicates total sample size.

Other notable ideas were that the government should make moves to increase birth in hospitals/health centers, either through encouragement or by building more infrastructure (8.7%). Looking across responses, a common theme was also the need to connect services directly to the community level. This idea was also commonly tied with the hiring, funding and dispatch of representatives charged with the task of visiting the community, school or household, and in order to check, register or educate every member of the community area. Answer to this question also showed significant connection to possession of a birth certificate. Chi square analysis (Table 4) showed that those who mentioned importance of enforcement had certificates 62.7% of the time. This was also true for the strategy of increasing hospital births (65.9% having certificates). On the other hand, those who mentioned the need for the government to take responsibility, or to reduce costs, were less likely to have a certificate (36.7 and 32%, respectively).

Finally, when asked how they themselves could encourage their parents to register or get certificates (Table 4), the majority of students mentioned that they would communicate some aspect of its importance as an impetus for parent action. Among such answers, 32.1% explicitly said that they would stress need of registration when taking school examinations, while others said they would stress importance for obtaining an ID or citizenship, future employment, or legal necessity. On the other hand, 9.7% said that they would arrange for their parents to talk with a third party (chief, head teacher), and 7.8% said that they would themselves act to pursue registration or directly ask their parents to accompany them. Chi square analysis of this question showed significant relation to possession of a certificate. Those who gave answers stressing general importance, asking for third party communication or taking action themselves had highest rates, while those who stressed tie to jobs or school were lower.

## Discussion

This study considered students’ awareness of the purpose and procedures for certification of their own births. This was pursued because these children represent the front line of the ongoing under-registration of children within developing regions, and will themselves soon be required to have birth certificates to continue schooling. Thus, it was argued that investigating what they know about registration, as well as awareness of their own rights, protections or empowerment, may afford an important addition to the understanding of the decisions of their community and parents. Our findings do provide important evidence that may help to further articulate this topic.

First, we actually found higher rates of certificate possession among students in this study, with just under half (43%) having birth certificates. While again care should be taken when considering these totals, this compares to only 8% of households in our previous study reporting all children with certificates, and 24.9% found in the 2008/09 Demographic and Health Survey [26] for the Coast Province region, of which Kwale is a part. The reason for this difference appeared to be the older age of this population, consisting of the three grades directly preceding the high school entrance examination. The students also showed low incidence of “Don’t know” responses, which can—in cases of high incidence, and in addition to simply not knowing—also indicate problems in survey methodology or question understanding, especially with children [17].

When we did look to the breakdown between grade levels there was significant difference in rates of students having certificates, with the highest totals among those in grade eight directly preceding examination. Students also showed much higher rates in the three years (2010–2012) before the study and falling off to almost no certification before this period. This trend of late registration also could be seen when asking about students’ younger siblings, who had certificates at a lower rate (34%).

Looking to other structural factors that correlated to whether or not children had certificates, we again found essentially the same issues as were found in our previous study and which also articulated a school-centered interpretation. For example, there were significant differences between Muslim and Protestant/Catholic populations, with the former showing lower rates, as well as between schools. These differences between religion as well as regions also coincided with similar findings in the adult population. However when we look at the breakdown between factors, no noticeable trends were discovered for any factor that would point to why children did not have certificates.

The same argument can be made for differences between schools. Although significant, consideration of other contextual aspects did not indicate any one reason that could be attributed to different rates of registration. In turn, when we look to our previous adult dataset we see that in fact there is not a correspondence between the highest registered schools and relative rates of registration in their wider regions. Rather, this appeared to be mainly tied to parent personal belief in importance of registration itself. This is also supported by significant correspondence between the students’ certification and the rates of certification of their younger siblings within the same household.

As discussed in our previous paper, this present situation whereby parents do not take action unless there is a pending need or positive benefit leads to the present issue witnessed in Kenya and many other developing regions of a significant time gap wherein children go without documentation. This same time period—in the present case roughly fourteen years—is also the ages whereby a child would most benefit from the health and social protections afforded by being recorded and identified by the government. Even more, despite need of birth certification for these children in order to continue their education, almost half still did not have a birth certificate by grade eight, foreshadowing the secondary issues endemic in developing regions from not having a record of children’s birth.

### Students’ awareness of issues affecting their possession of certificates

Regarding what children themselves know about registration or need of birth certificates, here the most noticeable finding may be the simple fact that children appeared highly informed regarding the need and procedures for securing a certificate. More than 90% could articulate a definition of a birth certificate, with the majority explaining that it contained vital and/or birth statistics.

Interestingly, this number of correct respondents was actually higher among students than their parents, where only 44.5% could articulate the definition of a certificate. Even more, most students (~93%) could definitively answer whether or not they themselves had a certificate, and could also answer for their siblings. Over half of students could also give examples of other children who had certificates. For comparison, our survey of parents showed that 13% responded that they did not know whether their children’s births had been registered, and 75% said that they did not know whether they had obtained a birth certificate. This difference might of course be interpreted as the product of a number of factors. It is possible that this reflects the, possibly unfounded, confidence of youth—note that we did not check whether they actually did have a birth certificate. Parents may also have had lower actual awareness than was found among children—a rather intriguing predicament. More plausibly, adults may have had higher embarrassment or unwillingness to give a candid answer, especially in negative registration cases [17], thus again pointing to the knowledge that might be gained from asking children about activity of their parents.

The majority (93%) also knew that certificates were necessary, while there also appeared to be preexisting official means of educating children about certificates, with children listing either chiefs—the local head of government—or schools and hospitals as an information source. Interestingly, this mention of health workers and local government as primary sources coincides with our finding among parents. This finding might also be interpreted to support the conclusion that only about 25% of students had received information from their parents or from their own experience of the births of their siblings, suggesting that there was not extensive parent-child communication regarding this topic.

When looking to answers regarding why one needed a certificate, there also appeared to be a lack of clarity, with a roughly even breakdown of answers between the given choices of either its basic legal requirement, its need in order to be recognized as a citizen, or for exam registration. This suggests a slight difference in understanding or awareness from parents, where most were able to articulate that registration or certification were necessary in order for identification or citizenship. However, among parents we also did find essentially the same number claiming the basic importance for school enrollment. Children also did not seem to know when one should get a certificate with even distribution between the given choices, and did not appear to have a strong awareness of who was responsible, with only a third claiming their parents. On the other hand, children did appear more knowledgeable about the procedure for obtaining a certificate, with the majority listing chiefs/assistant chiefs—the correct answer in most cases. The remainder mostly mentioned nurses or health attendants who also are traditional means of registration, especially in hospital births.

The findings raise another interesting angle regarding children and their parents. While children appeared to be informed about the basic need and purpose of registration, they did not appear highly knowledgeable about the role that should be played by their parents in securing registration. Our findings regarding knowledge or awareness questions might be taken as an indicator of an information gap in communication between parents, community and children when considering actual choice to pursue certification. Again, children without certificates were more likely to say that the purpose of a certificate was for school exam registration and less likely to frame its importance around citizenship or identification than were those presently with certificates. This might be read to suggest that children and potentially parents who had not certified were only now becoming aware of registration or certificate importance as a result of school involvement. This is also supported by our finding of significant difference in source of information, with those not registered mentioning schools as their primary source of information. While those who were registered were much more likely to mention chiefs or central government representatives, who presumably might frame registration importance along children’s rights, policy planning or citizenship. The same pattern was also found in our study of the adult population, in which a significant distribution was found regarding source of information and successful registration of all children, and where those parents who listed chiefs or health facilities registered at a much higher rate than those who listed schools as their primary source.

The above finding also leads to a handful of compelling questions. First, it may be that some communities had better established structures for communication or collaboration between citizens, government and children whereby they were made aware of the need for registration from the moment of birth. In addition, it is potentially important to discover whether the source of knowledge or awareness was the result of the actions of government representatives, parents or another third party. Alternatively, the finding of awareness of the chief as a source of information among children also raises the question of why these children became informed. That is, was this knowledge passed directly to children, or learned via their parents? An answer aligning especially to the former might suggest that having informed children, who can then better interact regarding this topic, may lead to quicker action by parents. In turn, these findings might also suggest that gains could be made from facilitating discussion between children and parents. Previous research shows that effective parent-child communication (for example regarding discussion of sexual issues) is associated with improved outcomes in children and adolescents [16], and might also work to motivate caregivers.

### What do students suggest?

The results also give important insights regarding how children suggest we might increase registration and possession of birth certificates. Beginning with the specific question of what could be done to motivate their parents, children most often argued for better articulation, to parents, of registration’s purpose or use. The factor that most children said they would mention was school registration, followed by identification, employment or basic importance. Interestingly, there also did appear to be a trend whereby children who were themselves not yet registered explicitly noted that they would stress the need of having a certificate for the purpose of citizenship or identification. This occurred at about double the rates of those who were not registered. At the same time, those who were already registered also noted more often that instead of communicating directly with parents they would put their parent in touch with an authority figure, chief or other government representative. These findings do suggest that children may have been quite aware of the underlying issues raised by our previous adult study and a prescient potential source for strategic planning.

When asked to give strategy ideas that could be used by the government, students again seemed quite in tune with the motivations or potential actions of their parents. Many argued for direct action from the government, and essentially for authorities to take the responsibility from parents. This was often framed along the lines of a need to fund, hire and send staff directly to the village, school, or the door of the parent. On the other hand, the other most often mentioned means were for officials to visit each village and provide more information, education, direct paperwork assistance and for them to ensure that each child was registered. This finding matches the argument made in our previous paper, where we suggested that the best course of action was for direct government involvement and action in place of relying on the motivation of the parent.

It is also informative to note what answers *were not* often given. While many students mentioned the need for building registration centers in their local areas or otherwise connecting services more directly to parents, only 7.8% argued for bureaucratic or structural developments to make the process more easy, and only 5% mentioned cost. As we discussed in our previous paper, while prevalent wisdom for increasing certification frames policy along the lines of cost or access for parents, the present low rates of registration even following programs to alleviate these issues suggests that this does not have the desired impact. Rather, the best case may be structural change that removes deliberation from the parent.

Children seemed to share this intuition. Equally interesting was our finding of some students (8.2%), again with no prompting, who explicitly mentioned that the government should increase birth in hospitals or build more health centers to improve rates of registration. This suggestion, which might provide an automatic means of registering and certifying children as part of the official course of hospital admittance, was given as a major suggestion from our previous paper. Our finding in this paper of likelihood among those students who were registered to suggest either hospitalization or direct enforcement, is further evidence for the intuition of children.

## Conclusion and Suggestions for Policy Planning and Research

We will conclude with a short discussion of potential application of these findings to policy or health research, and some suggestions. Although not necessarily representative of all children, these results do raise important implications for policy and study in Kenya, East Africa, or in similar developing regions. First, the results do specifically lend support to our previous arguments for policy implementations. We again had argued that a policy that can automate procedures or reduce responsibility or deliberation from parents may mark the most impactful solution. This is because parents in previously under-registered regions, *as reiterated here by their children*, do not appear motivated by the relative ease, cost or awareness of registration services. Rather they appear to take action only when they perceive an immediate benefit. In our present survey the contextual factors for when and why children were or were not registered appeared to center on parents waiting to register until it was immediately needed for school registration, and highlighting the need for different approaches that may minimize this decision.

In turn, when we look to what these children themselves suggested as possible solutions, a notable portion also argued for coupling registration to community centered programs that have immediate perceived benefit to themselves or their parents. Children also noted that such a strategy should include either directly registering a child for the parents, appointing individuals to visit each and every household in a village, or increasing hospital births. All three factors, especially entering children into a hospital system, were also stressed in our previous paper and do appear to be intuited by students.

Students also appeared quite cognizant of the deliberation and lack of sufficient motivation by their parents. Our respondents routinely stressed that parents do not find benefit until school becomes an issue, do not register younger children. They also suggested educational programs which stress earlier benefits such as need for child health or rights/identity protection.

Coupling children’s’ ideas to policy planning, one of the most obvious approaches would again be hospitalization for child birth, which was shown in our adult study to be the largest determinant of registration and to provide a structure allowing automatic registration services. Despite the advocacy of this approach also by a notable number of students, however, wide-scale hospitalization may not yet be practical in Kenya or other developing regions. Therefore we suggested again other structures that could provide similar automatic registration, such as child vaccination. As also noted in our last paper, this could also be accomplished through use of recent advances in ICT and mobile technology, which has shown promise in many similar areas [8, 34]. Interestingly, this structure for a representative to visit every home within a village in order to confirm or process registration was also an idea also specifically articulated by many children.

Second, when considering education services for informing parents, there also appears room for an important tweaking of present approaches. While it may seem intuitive to tie registration to schooling, because it would impact the most children even throughout rural or under-reported areas, this may be counterproductive. This is so because it appears to cause parents to wait until the last moment. As argued before, there may be more merit in reshaping education so that it does clearly articulate the immediate benefits, to children *and to parents*. This might even be done at the expense of downplaying latter educational or economic motives. This finding also appeared well intuited by children.

Finally, findings suggest that taking heed of ideas of children, especially as it pertains to the motivations of their parents, may itself play an important role in policy planning. They may be more candid or realistic sources for uncovering the actual factors that motivate the decisions or behaviors of the adult population. It should of course be explicitly noted that we are not advocating a shift of the burden for policy direction from adults to kids. Nor are we suggesting that this population of children in Kenya can speak for other countries’ children, who may of course have quite different circumstances.

The present study also does of course come with caveats. Children represent a challenging population for survey-based studies, especially regarding a tendency to not understand procedures or questions. See [15, 31, 35] for examples from Kenya. Although this study did use the utmost care in its procedures, following established practice for children, readers should be mindful when considering the findings. The study was also a convenience sample, albeit of a targeted population. Thus, care should be taken in making inferences regarding other children or other countries’ populations. We again in fact suggest against using this paper’s findings as de facto estimates of total rates—i.e., of birth certification. These are better addressed through our earlier adult study. However, especially in a case such as birth registration which does directly touch the lives of students, it might be argued that directly asking for their insights or opinions should at least be one aspect of research.
